# Supplementary material for: Analysis of Microbial Diversity and Community Structure of Rhizosphere Soil of Three Astragalus Species Grown in Special High-Cold Environment of Northwestern Yunnan, China
Source: Microorganisms. 2024 Mar 7;12(3):539. doi: 10.3390/microorganisms12030539 (PMC10975391; doi:10.3390/microorganisms12030539)
Supplement: Supplementary file 1 [file microorganisms-12-00539-s001.zip › Supplementary Figure S1 and Table S1.pdf]

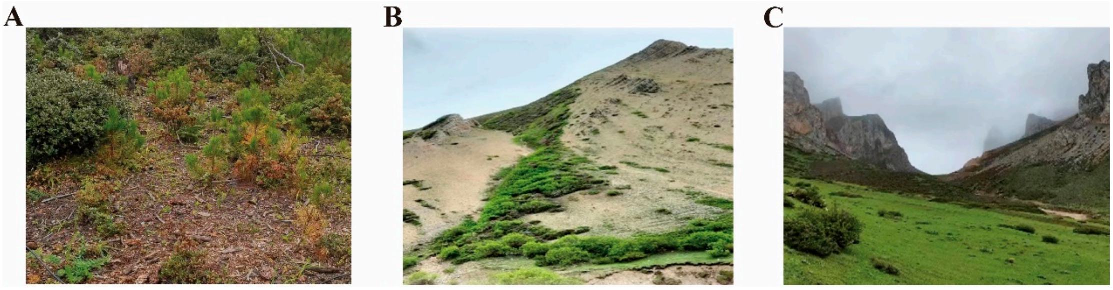

Figure S1

The plants of *Astragalus forrestii* (AF) is surrounded by more pine trees and deciduous foliage (A), *A. ernestii* (AE) grows in the steep slopes of flowstone beaches (B), and *A. acaulis* (AA) grows in alpine grassland (C).

Table S1 Information of samples collection for *Astragalus forrestii* (AF), *A. acaulis* (AA) and *A. ernestii* (AE)

Sample ID	Locality	Logitude/Latitude	Altitude(m)
AF	Dêqên	27°26′N 99°49′E	3,225
AA	Dêqên	28°19′N 99°6′ E	4,353
AE	Dêqên	28°19′N 99°6′ E	4,048
